# The Radical Cure of Aneurism

**Published:** 1907-01

**Authors:** 


### THE RADICAL CURE OF ANEURISM.

R. Matas (*Journal of the American Medical Association,* September 29, 1906) gives the history of his operation of intrasaccular suture, and remarks that in spite of his efforts to elucidate the general principles and method of the operation a good deal of misapprehension still seems to exist. He here describes the operation as consisting of two fundamental procedures: (1) The obliteration by suture of the vascular orifices which open into the aneurismal sac; (2) the obliteration of the sac by suture, which brings its inner surfaces in apposition, or by other methods which leave the sac undisturbed and tend to secure primary healing. In obliterating the vascular orifices that supply the sac, the parent trunk which nourishes the aneurism may be preserved or obliterated at the point of attachment, according to the type of sac encountered. Hence the subdivision into two varieties:

"1. Obliterative endoaneurismorrhaphy (the fundamental procedure) essentially consists in opening the sac freely without disturbing it from its surroundings, and closing all visible arterial orifices within the sac by suture, thus securing complete hemostatis and permanently stopping all further access of blood into the aneurismal cavity. The sac is obliterated by approximating its walls with buried sutures and closing the wound, with or without drainage; or, in rigid cavities, by simply infolding the overlaying skin flaps and lining the cavity with them (capitonnage of the French), or by one of the several procedures or variations I have suggested." This method is indicated in all aneurisms in which the sac is of a fusiform type in which there are two or more orifices of supply, and in which the parent artery is entirely lost at the seat of the aneurism by blending with the aneurismal sac throughout its circumference. In these cases no attempt is made to restore the continuity of the parent artery. The blood-stream is interrupted in that part af the vessel directly opening into the sac, to which all sources of blood-supply are cut off by suture. Twenty-two of the American cases reported are of this operation.

"2. Restorative endoaneurismorrhaphy. This variation in the procedure is solely applicable in aneurisms of the sacciform type, in which the parent trunk retains its continuity and normal outline, and the aneurism is a sac simply grafted on the vessel. By opening the sac freely and washing out the clot the opening leading to the artery is exposed inside the aneurism, and is readily closed by a continued suture which penetrates through all the coats of the sac at the margin of the orifice of communication. By this procedure the blood-supply of the sac is permanently arrested, the lumen of the parent artery remains patulous, and the arterial stream supplying the limb or dependent territory is immediately restored throuhg its normal channel. The sac is then obliterated by bringing its endothelial surfaces together with buried sutures, and the surface wound is closed in the usual manner. Up to the present time seven operations of this type have been reported by American surgeons, all successful, and two at least of the foreign cases may be appropriately added to this list, making nine in all.

"3. Reconstructive endoaneurismorrhaphy (arterioplasty) is applicable solely to fusiform aneurisms in which the coats of the sac are firm and resistant, and the two openings leading to the main artery lie on the same level, in close proximity, and are situated at the bottom of a superficial or readily accessible sac. In aneurisms of this type, especially those of traumatic origin, the continuity of the parent artery may be restored by building a new channel out of the sac walls, which can be brought together by suture over a guide (catheter or drainage-tube) inserted into the proximal and distal openings of the aneurism. Before tying the last sutures the guide is removed and the channel is left behind, corresponding to the outline of the original artery. The sac is then obliterated by approximating its surfaces with buried catgut suture, as previously applied in the first and second procedures."

Thus far five cases of this operation have been reported, all by American surgeons. Two of these patients have relapsed, and in one of these he thinks the amputation which followed might have been saved had a secondary obliterative operation been performed. A summary of the heretofore recorded and well-attested cases of these operations is given.—*Medical Age,* November 25, 1906.

